# Identification of NCAM that interacts with the PHE-CoV spike protein

**DOI:** 10.1186/1743-422X-7-254

**Published:** 2010-09-24

**Authors:** Wei Gao, Wenqi He, Kui Zhao, Huijun Lu, Wenzhi Ren, Chongtao Du, Keyan Chen, Yungang Lan, Deguang Song, Feng Gao

**Affiliations:** 1 College of Animal Science and Veterinary Medicine, Jilin University, Changchun 130062, PR China; 2 Key Laboratory of Zoonosis, Ministry of Education, Institute of Zoonosis, Jilin University, Changchun 130062, PR China; 3 Laboratory Animal Center, Jilin University, Changchun 130062, PR China

## Abstract

**Background:**

The spike proteins of coronaviruses associate with cellular molecules to mediate infection of their target cells. The characterization of cellular proteins required for virus infection is essential for understanding viral life cycles and may provide cellular targets for antiviral therapies.

**Results:**

We identified Neural Cell Adhesion Molecule (NCAM) as a novel interacting partner of the PHE-CoV S protein. A T7 phage display cDNA library from N2a cells was constructed, and the library was screened with the soluble PHE-CoV S glycoproteins. We used a coimmunoprecipitation assay to show that only the NCAM was a binding partner of spike protein. We found that a soluble form of anti-NCAM antibody blocked association of the PHE-CoV with N2a cells. Furthermore, double-stranded siRNA targeted against NCAM inhibited PHE-CoV infection.

**Conclusions:**

A novel interaction was identified between NCAM and spike protein and this association is critical during PHE-CoV infection.

## Background

Porcine hemagglutinating encephalomyelitis coronavirus (PHE-CoV) is a member of the Coronaviridae family, which causes porcine encephalomyelitis[1]. The mechanisms by which PHE-CoV infects cells and causes disease are not well characterized, nor are the factors known which determine the host and tissue specificity. The cellular receptor which is a crucial determinant of the tropism of several viruses, is not known in the case of PHE-CoV.

The spike glycoprotein of coronavirus is a major determinant of neurovirulence[2-5]. The coronavirus spike glycoprotein is responsible for viral attachment to the cellular receptor and fusion of the viral and cellular membranes, resulting in virus entry[4]. Several types of receptors for coronavirus have been previously identified[6]. The murine carcinoembryonic antigen cell adhesion molecule 1 (CEACAM1) and related murine glycoproteins in the carcinoembryonic antigen family of the Ig superfamily are the receptors for the murine coronavirus mouse hepatitis virus[4]. The aminopeptidase N (APN) glycoproteins are the receptors for human coronavirus 229E (HCoV-229E), the transmissible gastroenteritis virus of swine, and the feline coronavirus of genetic group 1[7-10].

PHE-CoV has a strong tropism for the central nervous system (CNS)[11]. The virus spreads via peripheral nerves to the CNS. PHE-CoV propagates mainly in the CNS, and nerve cells are a main target for virus replication[12]. The molecular mechanisms and specific proteins involved in adhesion of PHE-CoV to host cells have not yet been elucidated.

In this work, we discovered that the PHE-CoV S protein interacted with NCAM by screening a T7 phage cDNA library from Neuro-2a (N2a) cells. It is necessary to investigate these interactions with host-cell proteins, as discovering these interactions may be helpful in the identification of host proteins participating in important stages of the virus life cycle, such as virus entry, virion morphogenesis, and virion release. In addition, established protein contacts could serve as targets for antiviral chemotherapy.

## Methods

### Animals

Specific pathogen-free lines of piglets were purchased from the Centre for Medicine Animal Research (Jilin, China). Animal procurement and transportation into the HEPA-ventilated caging systems and performance of the experimental-challenge tests were performed in accordance with the guidelines for animal experimentation of Jilin University.

### Viruses and cell culture

The 67N strain of PHE-CoV[13] was propagated and assayed by the plaque method in N2a cell culture, as described previously[14], and the titres were expressed as plaque-forming units (PFU). The cell lines were obtained from the American Type Culture Collection (ATCC), N2a (ATCC CCL-131) and 293T (ATCC CRL-11268). These cells were maintained in Dulbecco's modified Eagle's medium (Invitrogen, Carlsbad, CA) supplemented with 10% cosmic calf serum (HyClone, Logan, UT) and 2 mM L-glutamine. All of the cell cultures were maintained at 37°C in 5% CO_2_.

### Protein production

The recombinant S protein of PHE-CoV was obtained using a Pichia pastoris yeast expression system. The S gene was subcloned by PCR. The forward primer for the S gene (5'-CGGAATTCGTGCCATCTATTAGCTCTGAAGT-3') and the reverse primer for the S gene (5'-TTGCGGCCGCAAGTATGCCCTGGCCTGTAATG-3') introduced EcoRI and NotI sites, respectively. Following gel purification, using the QIAquick gel extraction kit (Qiagen, Valencia, CA), the purified PCR products were ligated into the EcoRI and NotI sites of the pPICZαA vector (Invitrogen, San Diego, CA), yielding pPICZαAS. GS115 yeast cells, transformed with pPICZαAS (Invitrogen, San Diego, CA), were grown at 30°C in 100 ml liquid Buffered Methanol Complex Medium (BMMY) (Invitrogen, San Diego, CA) with 0.1 mg/ml Zeocine (Invitrogen, San Diego, CA). Production of the His6-tagged fusion S protein was induced with 1% methanol. After 5 d, the protein was collected from the supernatant. The His6-tagged recombinant S protein was purified by nickel affinity chromatography with the HisTrap HP column (Amersham Biosciences AB, Uppsala, Sweden).

### Preparation of the T7 phage display library from N2a cells

Total RNA from the N2a cells was extracted using standard methodology, while mRNA was purified using the poly (A) Quick mRNA Isolation Kit (Promega, Southampton, UK). A cDNA library was constructed with 10 μg mRNA, following the manufacturer's instructions for the OrientExpress Random Primer cDNA Synthesis kit (Novagen, Madison, WI), with some modifications. The first and second strand cDNA syntheses are simple reactions that are carried out sequentially in the presence of 5-methyl dCTP, which protects any internal EcoR I and Hind III restriction sites from digestion. The cDNA was treated with T4 DNA polymerase to blunt the ends, and EcoR I/Hind III Directional Linker was added at the end. Following, the cDNA fragments were digested with EcoRI and HindIII. The Mini Column Fractionation Kit (Novagen, Madison, WI) is used for rapid and effective size fractionation of DNA and removal of small molecules (< 300 bp) from DNA solutions by gel filtration. The cDNA fragments were ligated to the arms of T7 Select 10-3b and packaged in vitro using a T7 packaging extract (Novagen, Madison, WI), according to the manufacturer's directions. The packaged phage were amplified in liquid media with the host Escherichia coli BLT5403.

### Panning

In order to screen the clones that display the adhesion protein, the cDNA library from N2a cells was panned with the S protein. The 96-well plates were coated with 200 μl of the purified S protein (2 mg/ml) in coating buffer (50 mM NaHCO_3 _pH 9.6) overnight at 4°C. Nonspecific sites were blocked with 5% bovine serum albumin for 1 h at 37°C, and a 100 μl aliquot of the T7 phage display library (containing 6.4 × 10^10 ^PFU/ml) was added to the wells and incubated for 2 h at 37°C. Following this, the wells were washed five times with PBST (phosphate-buffered saline containing 0.1% [v/v] Tween-20) to discard any unbound phages. The bound phages were eluted with 200 μl of T7 elution buffer (TBS in 1% sodium dodecyl sulfate [SDS]) and amplified by infecting Escherichia coli BLT5403[15]. The amplified phages were then subjected to another four rounds of panning as described above, to enrich the clones that were highly specific for the S protein of PHE-CoV.

### Sequence analysis

After five rounds of panning, the final enriched specific clones were plated and single pure plaques were isolated. The cDNA inserts in these plaques were amplified by PCR using primers (T7 Select Up primer: 5'-GGAGCTGTCGTATTCCAGTC-3'; T7 Select Down primer: 5'-AACCCCTCAAGACCCGTTTA-3') flanking the inserts. Each PCR consisted of 30 cycles of denaturation at 94°C for 1 min, annealing at 50°C for 1 min, and extension at 72°C for 1 min. The reaction also included an initial denaturation step at 94°C for 5 min and a final extension step at 72°C for 7 min. After PCR amplification, the products were purified by Qiaquick columns (Qiagen, Hilden, Germany) and were then sequenced.

The nucleotide sequence of the protein that was most predominantly recognized by the S protein of PHE-CoV was a hypothetical gene of N2a cells http://www.ncbi.nlm.nih.gov/blast.

### Transfections and co-immunoprecipitation

The PHE-CoV 67N strain did not infect the 293T cell line. To investigate the interactions between the PHE-CoV 67N strain and the chimeric protein, 293T cells were transfected with the pcDNA3.1 (+) (Invitrogen, Carlsbad, CA) expression plasmid containing the chimeric gene, using Lipofectamine 2000 (Invitrogen, Carlsbad, CA). The transfections were performed following the manufacturers' protocols[16,17]. After 24 h, the cells were replated in selective media containing 50-100 μg/ml ampicillin[18], and single ampicillin-resistant clones were selected.

For co-immunoprecipitation, cells were lysed in 500 μl of radioimmune precipitation buffer (150 mm NaCl, 5 mg/ml sodium deoxycholate, 50 mm Tris-HCl, pH 7.5, 1% Nonidet P-40, 0.1% SDS) supplemented with freshly added protease inhibitors. After rotating for 1 h at 4°C, cell lysates were cleared by centrifugation at 8000 × g for 10 min at 4°C. The 100-ml aliquot of lysate was incubated with 3 ml of glutathione-Sepharose beads conjugated with His6-tagged fusion S (6 mg). Cell lysates were electrophoresed through 12% sodium dodecyl sulfate-polyacrylamide gels and transferred to polyvinylidene difluoride membranes. The blots were blocked at room temperature for 3 h with 3% BSA in PBS containing Tween 20 (0.05%) and then incubated overnight with a 1:2,000 dilution of the rabbit anti-S protein antibody. The blot were washed again and exposed to films[19].

### Flow Cytometry

We investigated whether a soluble form of the rabbit anti-NCAM antibody (Santa Cruz, California, USA, CATALOG: SC-10735) could inhibit PHE-CoV binding to N2a cells. The anti-NCAM antibody was diluted and added to N2a cells. The cells were incubated with 100 μl of soluble anti-NCAM antibody (10-25 μg/ml) for 1 h at 37°C. The controls included cells with goat IgG (1:1000) (Maixin, Fuzhou, China). Following this, the wells were washed five times with PBS (phosphate-buffered saline). After 2 hours, the PHE-CoV 67N strain (diluted to yield 20 to 40 plaques/well in 20 μl) was added to N2a cells that had been grown at a plating density of 10^5 ^cells per well in 24-well plates. After a 48-h infection, PHE-CoV binding was detected with the Rabbit PHE-CoV antiserum. The N2a cells were coated with 20 μl of rabbit anti-PHE-CoV antiserum at 1:1,000 per well for 1 h at 37°C. The cells were washed three times in PBS (pH7.4). Fluorescein (FITC)-conjugated goat anti-rabbit IgG (H+L) (Jackson ImmunoResearch Laboratory, West Grove, PA) was added to the N2a cell mixtures for 30 min. After 48 hours, the samples were analyzed on a BD FACSAria flow cytometer [6].

### Transfection of siRNAs and PHE-CoV infection

Double-stranded siRNA were designed based on the NCAM gene sequence to various regions of the genome using the Ambion siRNA Design tool http://www.ambion.com. Sequences were designed using (NN) N19 nt (where N is any nucleotide) and a GC content of less than 50%. The siRNAs targeted against the NCAM gene were synthesized at Sangon Biotech Co, Ltd. RNAs were deprotected and annealed using the Silencer siRNA Construction Kit (Ambions,Austin,USA). Double-stranded siRNA transfect into N2a cells using RNAimax (Invitrogen, Carlsbad,CA) as the transfection reagent. Before transfection, the cells were washed and resuspended in 900 μl of RPMI 1640 medium. Cationic lipid complexes, prepared by incubating 2 μM siRNA duplexes with 3 μl of oligofectamine in 100 μl of RPMI 1640 medium, were added to the wells. The effect of gene silencing was examined by indirect immunofluorescence. The resulting N2a cells were named N2a KD cells.

After a 24 h transfection, the PHE-CoV 67N strain was added to N2a KD cells. As control, the virus was added to mock-transfected siRNA N2a cells. At the indicated timings, culture supernatants were collected for plaque assay.

## Results

### Display of the cDNA library from N2a cells on T7 phage

**Figure 1:**
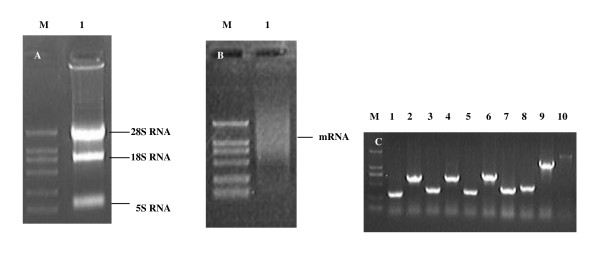
**The results of display of the cDNA library from N2a cells on T7 phage**. (A) Lane1:The result of extracted total RNA of N2a cells. The electrophoresis results show 28 S and 18 S bands were clear, indicating the total RNA extraction without degradation. M: DL2000 Marker. (B) Lane1:The result of purified mRNA of N2a cells. OD260/OD280 = 1.950. The data show that the purified mRNA could be used for cDNA synthesis. M: DL2000 Marker. (C) Lane1 to 10: The PCR identified result of randomly picked phage clones of the library. Amplification of inserts in randomly selected clones revealed that the library contained >90% recombinants with average insert size of >300 bp. M: DL2000 Marker.

T7 phage was enumerated using the plaque assay method on LB semi-solid medium. Based on the PFU after in vitro packaging, the T7 phage display library from the N2a cells was calculated to contain 1.5 × 10^7 ^independent clones. The amplified library with a titer of 6.4 × 10^10 ^pfu/mL was used for the subsequent screening. Amplification of the inserts in randomly selected clones revealed that the library contained >90% recombinants, with an average insert size of >300 bp. Because the size of the phage display library exceeded the estimated number, most of the expressed genes were represented in this library (Fig. 1).

### Affinity selection and sequence analysis of specific genes recognized by the S protein

The entire screening process was repeated for five rounds. After each round of panning, there was an increase in the number of clones, suggesting that the procedure enriched for specific clones (Table 1). By the end of the fifth round of panning, there was a 320-fold increase in specific clones compared to the number of clones that were obtained after the first round. However, there was no further enrichment after additional rounds of panning.

**Table 1:** Phage enrichment results after different rounds of panning.

Round of panning	Phage applied (PFU/ml)	Phage eluted (PFU/ml)	Enrichment (fold)
1	1.6 × 10^10^	5.5 × 10^5^	3.4 × 10^−5^
2	2.8 × 10^10^	5.3 × 10^6^	1.9 × 10^−4^
3	2.5 × 10^10^	8.3 × 10^6^	3.3 × 10^−4^
4	3.4 × 10^10^	5.1 × 10^7^	1.5 × 10^−3^
5	3.8 × 10^10^	6.5 × 10^7^	1.7 × 10^−3^

**Figure 2:**
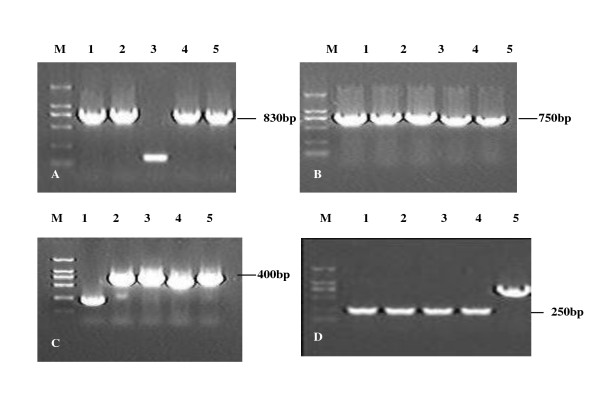
**Detection of inserted fragments of phage clones in the fifth round of selection library by PCR**. M: DL2000 Marker; Lane 1 to 5: The PCR result of randomly picked phage clones of the fifth round of selection library. (A) Approximately 38% of the phage clones had an insert size of 830 bp. (B) 25% of the phage clones had an insert size of 750 bp. (C) 22% of the phage clones had an insert size of 400 bp. (D) 15% of the phage clones had an insert size of 250 bp.

Approximately 100 clones were randomly picked from individual plaques, and the DNA sequences of clones were amplified by PCR and analyzed on an agarose gel to determine the insert size. Approximately 38% of the phage clones had an insert size of 830 bp, 25% had an insert size of 750 bp, 22% had an insert size of 400 bp and 15% had an insert size of 250 bp (Fig. 2). The clones were then further sequenced.

DNA sequences of the inserts from the fifth round of panning were determined and compared using BLAST analysis. Panning yielded four clones(Table 2), as follows: neural cell adhesion molecule (NCAM), splicing factor 3b, subunit 2 (Sf3b2), histone deacetylase 2 (Hdac2), and ribosomal protein S13 (RPS13).

**Table 2:** BLAST analysis identification of fifth round-insert sequences.

Clone name	**GenBank no**.	Identity
NC-1	NM_001081445	neural cell adhesion molecule (NCAM)
NC-2	NM_030109	splicing factor 3b, subunit 2 (Sf3b2)
NC-3	NM_008229	histone deacetylase 2 (Hdac2)
NC-4	NM_026533	ribosomal protein S13 (RPS13)

### Expression of NCAMSf3b2, Hdac2 and RPS13

**Figure 3:**
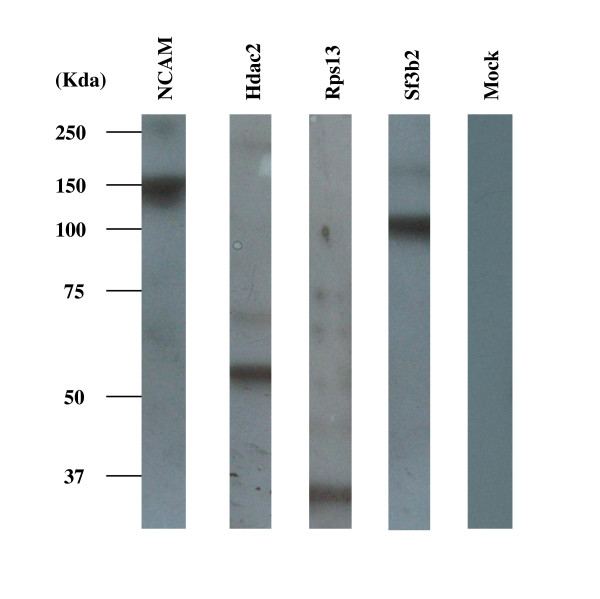
**Western blot analysis of proteins expression in total extracts of 293T cells transfected with the pcDNA3.1 (+) expression plasmid**. Lane 1: Western blot analysis of NCAM protein expression. The full lengths cDNA of NCAM gene was used to construct the transfect plasmid. Cell lysates from 293T cells were run on a 10% SDS-PAGE gel and blotted onto polyvinylidene difluoride membranes. The blots were probed with a 1:10 dilution of the rabbit anti-NCAM polyclonal IgG (200 μg/ml). The antibodies were detected by horseradish peroxidaseconjugated goat anti-rabbit IgG antibodies and chemiluminescence. Lane 2: Immunoblots for Hdac2 protein. The blot was probed with a 1:10 dilution of the rabbit anti-Hdac2 polyclonal IgG (200 μg/ml). The antibodies were detected by horseradish peroxidaseconjugated goat anti-rabbit IgG antibodies. Lane 3: Immunoblots for RPS13 protein. The blot was probed with a 1:10 dilution of the goat anti-RPS13 polyclonal IgG (200 μg/ml). The antibodies were detected by horseradish peroxidaseconjugated mouse anti-goat IgG antibodies. Lane 4: Immunoblots for Sf3b2 protein. The blot was probed with a 1:5 dilution of the mouse monoclonal anti-Sf3b2 IgG2a (100 μg/ml). The antibodies were detected by horseradish peroxidaseconjugated goat anti-mouse IgG antibodies. Lane 5: The 293T cells transfected with vector alone.

The full lengths cDNA of these genes (NCAM: GenBank no. NM_001081445Sf3b2: NM_030109, Hdac2: NM_008229 and RPS13: NM_026533) was used to construct the transfect plasmid. These four protein expression levels were detected by a BioPhotometer Plus (Eppendorf, Hamburg, Germany). The correct expression of NCAM, Sf3b2, Hdac2 and RPS13 in 293T cells was studied by immunoblotting. The four proteins antibodies were purchased from Santa Cruz biotechnology,inc (NCAM antibody CATALOG: SC-10735; Sf3b2 antibody: SC-101133; Hdac2 antibody: SC-7899; RPS13 antibody: SC-162098). The polypeptides migrated to a molecular weight corresponding to NCAM (140 kDa), Sf3b2 (100 kDa), Hdac2 (55 kDa) and RPS13 (17 kDa), respectively (Fig. 3).

### Identification of NCAM as a binding partner of the S protein

**Figure 4:**
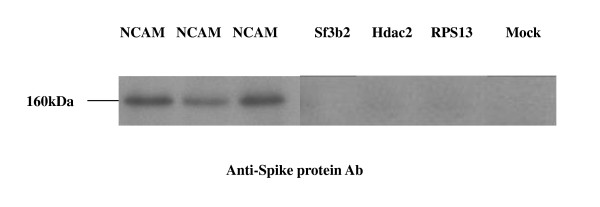
**The NCAM binding to PHE-CoV S protein**. Lane 1-3, NCAM involves in recognition by PHE-CoV S protein. Supernatants of 293T cells transfected with plasmid encoding soluble NCAM. The 293T cells were added fusion S protein (6 mg) and incubate for 2 h at 4°C. The cells were lysed in 500 μl of radioimmune precipitation buffer. The 10-ml aliquot of lysate was incubated with 300 μl of glutathione-Sepharose beads conjugated with fusion anti-S protein antibody and gently rocking on a orbital shaker overnight at 4°C. The sepharose beads are boiled for 5 min to dissociate the immunocomplexes from the beads. The supernatant was electrophoresed through 12% sodium dodecyl sulfate-polyacrylamide gels and transferred to polyvinylidene difluoride membranes. The blots were blocked at room temperature for 3 h with 3% BSA in PBS containing Tween 20 (0.05%) and then incubated overnight with the anti-NCAM protein antibody. The proteins was analyzed by western blotting. Lane 4-6, Sf3b2, Hdac2 and RPS13 were not immunoprecipitated with S protein. Lane 7, 293T cells transfected with vector alone were negative controls.

To identify the binding partner of S protein, co-immunoprecipitation was performed. The 293T cell lysates were immunoprecipitated with anti-S protein antibody. Supernatants of 293T cells transfected with plasmid encoding the screened gene were immunoprecipitated with S protein and anti-S protein antibody. The 293T cells transfected with vector alone were controls. When the soluble form of NCAM was incubated with S protein, a 160 kDa band was observed (Fig. 4). However, Sf3b2, Hdac2 and RPS13 were not immunoprecipitated with S protein. Moreover, the PHE-CoV spreads via peripheral nerves to the central nervous system. Sf3b2, Hdac2 and RPS13 are all expressed in various tissues and cells. Therefore, we did not analyze them further. The data demonstrate a specific, high-affinity association between the S protein of PHE-CoV and NCAM.

### Anti-NCAM antibody inhibit binding of PHE-CoV to N2a cells

**Figure 5:**
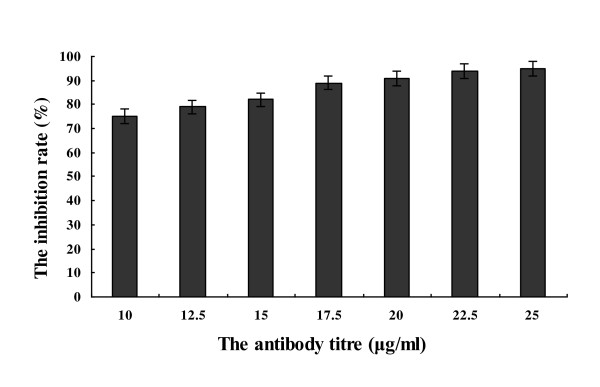
**Anti-NCAM antibody inhibition of PHE-CoV binding to N2a cells**. PHE-CoV binding assay using various concentrations of anti-NCAM antibody. The 10 μg/ml anti-NCAM antibody inhibited PHE-CoV binding to N2a cells by 75%. With the increased anti-NCAM antibody concentration in the blocking, the inhibition rate increased accordingly. The 25 μg/ml anti-NCAM antibody inhibited PHE-CoV binding to N2a cells by 95.7%. However, the proliferation of virus could not be suppressed completely.

We investigated whether anti-NCAM antibody could block the association of PHE-CoV with N2a cells. Virus binding was detected using PHE-CoV antiserum and FITC-conjugated goat anti-rabbit IgG (H+L). FACS analysis showed that the binding rate of PHE-CoV to N2a cells with control goat IgG was 99%. However, the 10 μg/ml anti-NCAM antibody inhibited PHE-CoV binding to N2a cells by 75%. With the increased anti-NCAM antibody concentration in the cell blocking, it was noticed that, the inhibition rate reached about 95% (Fig. 5). However, the proliferation of virus could not be suppressed completely. The result was that a soluble form of the anti-NCAM antibody blocked the association of PHE-CoV with the N2a cells.

### The NCAM siRNAs inhibit PHE-CoV infection for prolonged periods of time

**Figure 6:**
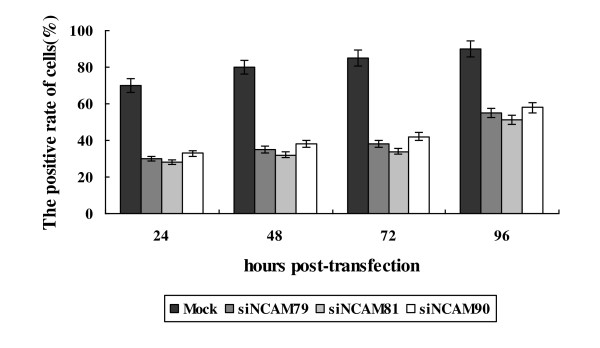
**Double-stranded siRNA could effectively inhibit NCAM expression in N2a cells**. N2a cells were transfected with siRNA targeted against NCAM. The cells were harvested after siRNA transfection and analyzed by FACS with rabbit anti-NCAM antibody and FITC-conjugated goat anti-rabbit IgG (H+L). Mock-transfected siRNA N2a cells served as a control. There appeared to be a slight decrease of the positive rate of N2a KD cells compared to that of controls within 72 hours.

**Figure 7:**
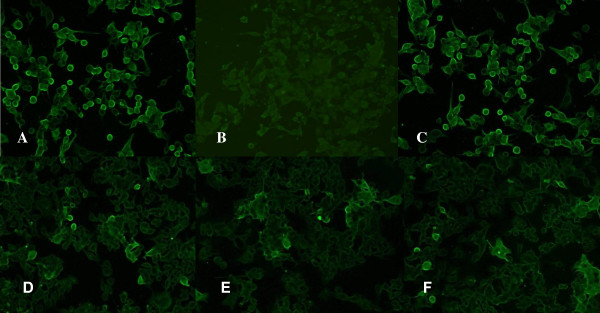
**The NCAM siRNAs inhibit PHE-CoV infection by indirect immunofluorescence**. After a 48 h viral infection, the N2a cells were fixed with 80% acetone for 10 min at -20°C, rehydrated in PBS, labeled with rabbit PHE-CoV antiserum, and washed three times with PBS. FITC-conjugated goat anti-rabbit IgG (H+L) (1:50 dilution) was added to the N2a cell mixtures for 30 min at room temperature, and the cells were washed and observed with an Olympus FV1000 laser scanning confocal microscope. Microscopic magnification, 400×. (A) Mock transfection (stained with PHE-CoV-positive serum); (B) Mock transfection (stained with PHE-CoV-negative serum); (C) siCtrl transfection; (D) siNCAM79 transfection; (E) siNCAM81 transfection; (F) siNCAM90 transfection.

**Figure 8:**
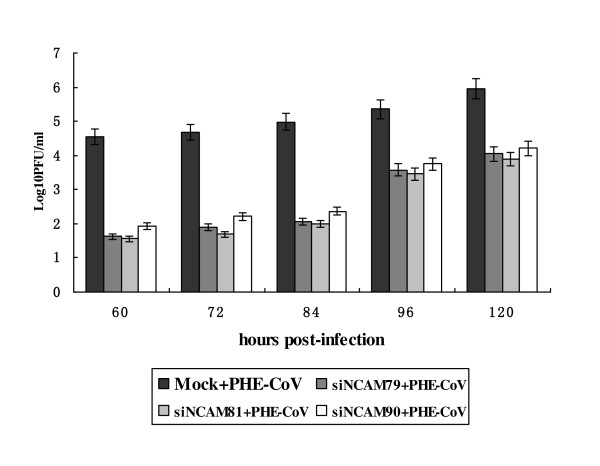
**The NCAM siRNAs could inhibit PHE-CoV infection in a period of time**. Culture supernantants were collected 120 hours after the PHE-CoV challenge. The supernatants harvested at indicated timings were subjected to plaque assay. There was significant difference (p < 0.05) in virus titres. Knock-down of NCAM caused a marked reduction of PHE-CoV infection within 84 hours.

All oligonucleotide sequences used to produce NCAM siRNA are shown in Table 3. Three siRNAs were designed based on the NCAM sequence (Accession no. NC_000075). The effect of NCAM gene silencing in N2a cells was confirmed by flow cytometry. The NCAM protein expression was completely suppressed within 72 h (Fig. 6). To determine the antiviral effects of siRNAs, N2a cells were transfected with NCAM siRNAs and challenged with the PHE-CoV 67N strain 24 hours later. The effects of the siRNAs in N2a cells stained after transfection and infection with PHE-CoV was analysed by indirect immunofluorescence (Fig. 7). After further culture for 5 days, the reduction of cell-free viral particle production was assessed by plaque assay. Plaque assay analysis of the cultures after infection revealed a corresponding reduction in siRNA-transfected N2a cells. The NCAM siRNAs inhibited PHE-CoV infection compared to controls throughout the 84-hour period of observation (Fig. 8). These results demonstrated that expression levels of NCAM correlate with PHE-CoV infection. NCAM might participate in the attachment and invasion of N2a cells.

**Table 3:** Oligonucleotides for siRNA construction.

siRNA	strand	Sequence
SiNCAM79	Antisense	5'-AAGGTCTTTGCAAAGCCCAAACCTGTCTC-3'
	Sense	5'-AATTTGGGCTTTGCAAAGACCCCTGTCTC-3'
SiNCAM81	Antisense	5'-AAGTCTATGTGGTAGCTGAAACCTGTCTC-3'
	Sense	5'-AATTTCAGCTACCACATAGACCCTGTCTC-3'
SiNCAM90	Antisense	5'-AACTCTGTCGAACCTCACAAACCTGTCTC-3'
	Sense	5'-AATTTGTGAGGTTCGACAGAGCCTGTCTC-3'
siCtrl	Antisense	5'-AATTTGGGCTTTGCAAAGACCTTCCTGTCTC-3'
	Sense	5'-AATTCCAGAAACGTTTCGGGTTTCCTGTCTC-3'

## Discussion

In this report, we describe the discovery of a novel interaction between NCAM and spike protein of PHE-CoV. To our knowledge, this is the first study that used a phage display-based cDNA expression library for screening and affinity panning with the PHE-CoV spike protein to identify the interaction between PHE-CoV and N2a cells. Co-immunoprecipitation analysis showed that the NCAM was a binding partner of spike protein. In addition, FACS analysis demonstrated that a soluble form of the anti-NCAM antibody blocked association of PHE-CoV with N2a cells. Moreover, double-stranded siRNA targeted against NCAM inhibit PHE-CoV infection. The results suggest that NCAM might participate in virus infection.

Neural Cell Adhesion Molecule (NCAM, also the cluster of differentiation CD56) is a homophilic and heterophilic binding glycoprotein expressed on the surface of neurons, glia, skeletal muscle and natural killer cells[20]. NCAM is a member of the immunoglobulin super-gene family of Cell adhesion molecules (CAMs)[21]. CAMs play important roles in cell-cell and cell-extracellular matrix interactions in both mature and developing nervous system[22]. During development, they are involved in cell migration, axon guidance, target recognition, and synapse formation; while in the mature nervous system, they maintain synaptic connections, cell-cell contacts, and neuron-glial interactions[22]. Injuries to the nervous systems break the stable state of the tissues and the repair of damaged tissues and regeneration of axons require the participation of CAMs both as adhesion molecules and as signal transduction molecules[22]. NCAM has been implicated as having a role in cell-cell adhesion, neurite outgrowth, synaptic plasticity, and learning and memory[23,24]. There is evidence that PHE-CoV is disseminated throughout the central nervous system by direct transfer of virus from neuron to neuron[25]. Thus, by binding to NCAM, the PHE-CoV might increase the probability of gaining access form peripheral nervous system to the central nervous system.

Affected piglets show the clinical symptoms such as generalized muscle trembling, abnormal walking, lack of co-ordination, ears held back, convulsions and lying on the side and paddling legs. If PHE-CoV bind to NCAM, certain aspects of the clinical symptoms may be readily explained. NCAM is expressed in the surface of developing muscle with a spatiotemporal pattern that is consistent with a role in neuromuscular junction (NMJ) formation[26]. Only NCAM of the CAMs appears on the surface of muscle cells in parallel with the ability of the muscle cell surface to accept synapses[27]. Levels of NCAM in muscle are regulated in parallel with the susceptibility of muscle to innervation. NCAM-induced sprouting is thought to be induced via homophilic binding between NCAMs in the neural and the muscle surfaces, that in turn induces growth promoting mechanisms in the nerve process[26].

The close genetic and antigenic relatedness among the group 2 coronaviruses human coronavirus OC43 (HcoV-OC43), bovine coronavirus (BCV), and porcine hemagglutinating encephalomyelitis virus (PHE-CoV) suggests that these three viruses with different host specificities diverged fairly recently[1]. HcoV-OC43, BCV and PHE-CoV recognize sialic acid-containing receptors similar to those of influenza C viruses[28-32]. Polysialic acid (PSA) is a developmentally regulated carbohydrate composed of a linear homopolymer of a-2,8-linked sialic acid residues[33]. NCAM undergoes post-translational modification during development, leading to the abundant addition of PSA chains on its extracellular domain[34]. PSA on NCAM is developmentally regulated thus playing a prominent role in different forms of neural plasticity spanning from embryonic to adult nervous system, including axonal growth, outgrowth and fasciculation, cell migration, synaptic plasticity, activity-induced plasticity, neuronal-glial plasticity, embryonic and adult neurogenesis[35].

The entry of coronaviruses is a multi-step process that involve: docking on the plasma membrane, binding to a receptor or co-receptors and delivery of the viral genome into the host cell. Docking of viruses on the plasma membrane of a susceptible cell is the first step during virus entry. Docking involves non-specific interactions between the viral envelope protein and carbohydrate moieties like heparan sulfate or sialic acid on the surface of cells. These initial docking interactions may lead to concentration of virus at the plasma membrane of a susceptible cell that in turn may enhance the infectivity of the virus by facilitating the interactions of the envelope protein with a cellular receptor that promotes virus entry. Reovirus strains that have sialic acid-binding activity attach to cells with 5-fold more avidity than strains that do not bind sialic acid, and their infectivity is enhanced 50-100 fold[36]. After docking at the surface of a susceptible cell, the virus binds a receptor molecule(s) that in turn triggers conformational changes that result in virus entry. We speculate that the entry of PHE-CoV is a multi-step process. The Hemagglutinin-esterase (HE) protein of PHE-CoV binds to polysialic acid (PSA) moieties, while the spike (S) protein of PHE-CoV binds to NCAM at the plasma.

Additionally, porcine hemagglutinating encephalomyelitis is an infectious disease affecting mainly pigs under 3 weeks old[37]. During the embryonic development of the brain, NCAM undergoes posttranslational modifications leading to the addition of a-2,8-polysialic acid (PSA) chains on its extracellular domain[38]. This embryonic highly PSA-NCAM is expressed abundantly throughout the brain until early postnatal period and is involved in neurite extension and synaptogenesis[38]. In the adult brain, however, PSA-NCAM expression is considerably reduced, although it has been shown to be expressed in certain areas (e.g. the olfactory bulb and hippocampus) [34].

Finally, identification of the NCAM that interacts with PHE-CoV spike protein will facilitate the description of the binding domain of the spike protein, which will presumably be the most effective target epitope for a spike protein-based subunit vaccine. In addition, it is likely that a cell line approved for vaccine production, and one that is made permissive for viral replication through expression of NCAM, will be the most efficient large-scale producer of whole-killed or attenuated virus for use as a vaccine. There are a number of chronic neurologic diseases, such as myasthenia gravis, subacute sclerosing panencephalitis, and Alzheimer's disease, for which some evidence of viral etiology exists [39]. One explanation for these diseases is that after a virus binds to a cellular constituent acting as a receptor, the receptor might be altered. Identification of the specific neuronal constituents to which neurotropic viruses bind will allow for an analysis of the potential effects of these interactions on functional or antigenic alterations of receptors [40].

## Competing interests

The authors declare that they have no competing interests.

## Authors' contributions

WG and WH carried out most of the experiments and wrote the manuscript. HL participated in the protein production. KZ carried out the co-immunoprecipitation assay. WR and CD participated in the sequence alignment. YL participated in the design of the NCAM siRNAs. KC participated in the design of the study. FG and DS conceived of the study and participated in its design and coordination. All authors read and approved the final manuscript.
